# The maize gene *ZmSBP17* encoding an SBP transcription factor confers osmotic resistance in transgenic *Arabidopsis*

**DOI:** 10.3389/fpls.2024.1483486

**Published:** 2024-11-07

**Authors:** Lifang Sun, Lijiao Wang, Jinping Niu, Wei Yang, Zhifang Li, Libin Liu, Shuren Gao

**Affiliations:** ^1^ Key Laboratory of Modern Agricultural Cultivation and Crop Germplasm Improvement of Heilongjiang Province, Agronomy College of Heilongjiang Bayi Agricultural University, Daqing, Heilongjiang, China; ^2^ Key Laboratory of Low Carbon Green Agriculture in Northeast Plain, Ministry of Agriculture and Rural Affairs, Daqing, Heilongjiang, China

**Keywords:** maize (Zea mays L.), osmotic stress, regulatory mechanism, SBP transcription factor, *Arabidopsis thaliana*

## Abstract

Among the major abiotic stresses, salt and drought have considerably affected agricultural development globally by interfering with gene expression profiles and cell metabolism. Transcription factors play crucial roles in activating or inhibiting the expression of stress-related genes in response to abiotic stress in plants. In this study, the Zea mays L. SQUAMOSA promoter-binding protein gene (ZmSBP17) was identified, and the molecular regulatory mechanism of osmotic stress tolerance was analyzed. Phylogenetic analysis confirmed that ZmSBP17 is part of the SBP gene family and is closely related to OsSBP17. The ZmSBP17-GFP fusion protein exhibited green fluorescence in the nucleus, as determined via tobacco epidermal transient transformation system. Acting as a transcriptional activator, the overexpression of ZmSBP17 in Arabidopsis significantly enhanced the expression of genes encoding superoxide dismutases (CSD1/2, MSD1), catalases (CAT1/2), ascorbate peroxidase 1 (APX1), and myeloblastosis transcription factors (AtMYB53/65), which increased the activity of reactive oxygen species (ROS)-scavenging enzymes and reduced ROS levels. Additionally, the expression of abiotic stress-related genes, such as AtDREB2A and AtNHX1, was significantly upregulated by ZmSBP17. Furthermore, ZmSBP17 specifically bound to cis-acting elements containing GTAC core sequences in the promoters of stress-related genes, suggesting that ZmSBP17 regulates the transcription of certain genes by recognizing these sequences. These results indicate that the overexpression of ZmSBP17 in Arabidopsis thaliana significantly increased tolerance to osmotic stress during the germination and seedling stages, which may enhance our understanding of the biological functions of SBPs in maize under abiotic stresses.

## 1 Introduction

Salinization of soil increases ion toxicity, making it difficult for roots to absorb water and nutrients, leading to physiological drought and nutritional deficiencies (Zhao et al., 2020). Saline–alkali soil, an important soil type, is widely distributed in over 100 countries. The Songnen Plain in Northeast China is a key grain production area and commodity grain base, and it is the primary region in China with saline–alkali soil. Additionally, drought, particularly spring drought, is a major abiotic stress prevalent in this region. Various abiotic stresses, including salt, drought, and cold, can impose osmotic stress on crops (Vinocur and Altman, 2005). Maize (*Zea mays* L.), a critical global food crop, is used for human consumption, animal feed, biofuel production, and other industrial purposes (Serna-Saldivar and Carrillo, 2018). Therefore, to ensure national food security and sustainable agricultural development, it is crucial to focus on the molecular basis of maize’s response to osmotic stress.

Transcription factors (TFs) are crucial in regulating the abiotic stress signal transduction pathway because they can activate or inhibit the expression of stress-related genes by interacting with their cis-elements (Agarwal et al., 2017). The SQUAMOSA promoter-binding protein (*SBP*) family is one of the largest plant-specific TF gene families. Members of this family contain a highly conserved DNA-binding domain known as the SBP-box (Klein et al., 1996), which consists of approximately 76 amino acid residues forming two zinc finger motifs (C3H and C2HC) and a nuclear localization signal (Yamasaki et al., 2013). SBP TFs have been identified in many plants, including *Arabidopsis thaliana* (Cardon et al., 1999), *Brassica napus* (Cheng et al., 2016), *Petunia* (Zhou et al., 2018), and wheat (Li et al., 2020). These TFs are known to be involved in regulating growth, development, and responses to abiotic stress. For example, in *A. thaliana*, *AtSPL3/8* influences floral development, with its expression induced by prolonged sunlight starting to increase before flowering (Unte et al., 2003; Cardon et al., 1997). *AtSPL10/11/12* redundantly controls the proper development of lateral organs in relation to shoot maturation during the reproductive phase (Shikata et al., 2009). In rice, *OsSPL10* negatively regulates salt tolerance but positively affects trichome formation (Lan et al., 2019), whereas *OsSPL3/12* positively regulates crown root formation (Shao et al., 2019). Overexpression of *VpSBP16* from grapes in *A. thaliana* significantly increases root length and seed germination rate under salt and drought stress (Hou et al., 2018). *VpSBP5* contributes to resistance against *Erysiphe necator* by inducing salicylic acid and methyl jasmonate signaling pathways (Hou et al., 2013). Mao et al. (2016) provided a comprehensive overview of SBP TFs in maize, noting that the expression of certain SBP TFs increased significantly under abiotic stress. However, the biological functions of SBPs in maize under abiotic stresses remain unclear.

In this study, a novel SBP transcription factor gene, *ZmSBP17*, was isolated from the inbred line Hei maize. Overexpression of *ZmSBP17* enhanced the plant’s tolerance to osmotic stress by increasing the expression of abiotic stress-related genes. The core GTAC sequence was found to be crucial for regulating gene expression mediated by *ZmSBP17*. This study enhances our understanding of the roles of maize SBP TFs and the mechanisms underlying plant tolerance to osmotic stress.

## 2 Materials and methods

### 2.1 Plant and osmotic stress treatments

Pre-germination treatments of maize seeds were performed and growth conditions applied as described in our previous study (Sun et al., 2018). Germinated seeds were sown in 20 × 22 cm plastic pots containing 2.5 kg vermiculite. All pots were watered with a half-strength Hoagland nutrient solution once daily. At the 3-leaf seedling stage of maize, 24 pots were selected and randomly divided into two equal sets. One set was used as a control by watering with a half-strength Hoagland nutrient solution, and the other set was treated with 200 mmol/L NaCl or 10% polyethylene glycol (PEG6000) in half-strength Hoagland nutrient solution. Leaves were harvested at 0, 2, 6, and 12 h post-stress treatment for RNA extraction to detect the expression pattern of *ZmSBP17* under abiotic stress. Three biological samples (three pots for one time in one set) were collected from each tissue.

*A. thaliana* Columbia ecotype was used in this study. Growth conditions in soil and stress treatment methods were applied as described by He et al., 2019, 2024. Four-week-old gene-overexpressing *Arabidopsis* lines (OE) and wild-type (WT) plants were treated with water (control) or 200 mmol/L NaCl and 10% PEG6000 for 36 h. Then, leaves were collected to analyze the expression levels of abiotic stress-related genes and determine the activities of key reactive oxygen species (ROS)-scavenging enzymes.

### 2.2 Real-time quantitative RT-PCR analysis

Total RNA was extracted from the samples using the RNAiso Reagent Kit (TaKaRa, Dalian, China). The FastQuant RT Kit (TIANGEN, China) was used to synthesize the first-strand cDNA, following the procedure outlined in our previous studies (Sun et al., 2012; 2018). *ZmActin1* (NM_001155179) was used as the endogenous control to compare the expression level of *ZmSBP17*. *ACT8* (*AT1G49240*) was utilized as the internal control to analyze the expression levels of abiotic stress-related genes in *Arabidopsis*. All primers used for qRT-PCR assay are listed in **Supplementary File 1**. A standard SYBR^®^ Premix Ex Taq™ kit (TaKaRa, Dalian, China), Bio-Rad CFX96 Real-Time System (Bio-Rad, USA, Hercules, CA, USA), and CFX Manager System software version 2.0 were utilized per the manufacturer’s instructions. At the end of PCR, Ct values for each sample were obtained to analyze the transcript levels of each gene using the 2^−ΔΔCt^ method (Livak and Schmittgen, 2001).

### 2.3 Gene isolation and sequence analysis of *ZmSBP17*

Based on the sequence of *ZmSBP17* available in the NCBI (http://www.ncbi.nlm.nih.gov/Blast.cgi) and maize genome (www.plantgdb.org/ZmGDB/cgi-bin/blastGDB.pl) databases, gene-specific primers for RT-PCR were designed using the NCBI primer designer. The primer sequences are listed in **Supplementary File 1**. Following the procedure outlined in our previous study (Sun et al., 2012), the full-length coding sequence (CDS) of Hei *ZmSBP17* was isolated from the first-strand cDNA, cloned into the pMD18-T cloning vector (TaKaRa, Dalian, China), and sequenced (BGI, China). NCBI’s Conserved Domain database (http://www.ncbi.nlm.nih.gov/Structure/cdd/wrpsb.cgi) was used to analyze the sequences and identify the conserved regions. Phylogenetic trees of ZmSBP17 and sequences from homologous plants were then constructed using DNAMAN software.

### 2.4 Subcellular localization, trans-activation, and DNA-binding assays of *ZmSBP17*

The full-length CDS of *ZmSBP17*, lacking its termination codon, was inserted into the pCAMBIA2300-GFP vector using a combination of homologous recombination and restriction digestion (*Sac*I and *Xba*I). This construct was then used to transform the *Escherichia coli* strain DH5α. Expression of *ZmSBP17* was driven by a CaMV 35S promoter. The primer sequences used for amplification are listed in **Supplementary File 1**. The validated recombinant pCAMBIA2300-*ZmSBP17*-GFP and pCAMBIA2300-GFP (control) vector were transformed into EHA105 *Agrobacterium* competent cells for injecting into 5-6 leaf old *N. benthamiana* leaves epidermal cells, respectively, as previously reported (Chao et al., 2023). The transformed tobacco plants were incubated at room temperature in the dark for 12-16 h, and then imaged by laser scanning confocal microscopy (Leica SP8) after cultured for 2-3 days at normal condition.

To detect the transcriptional activation of *ZmSBP17*, the full-length and different truncated versions, which were amplified (primers listed in **Supplementary File 1**) from the pMD18-T-*ZmSBP17* plasmid into the pGBKT7 vector. These constructs, along with the pGBKT7 control vector, were used to transform the yeast strain Y2HGold according to the manufacturer’s protocol (Clontech, USA). The transformants were then cultured on SD/-Trp and SD/-Trp/-His media for 3–5 days at 30°C.

For DNA-binding assays, the full-length *ZmSBP17* CDS was amplified and cloned into the pGAD7 vector. The sequences of the SBP17-F4 and R4 primers, which contain *Nde*I and *EcoR*I sites, are listed in **Supplementary File 1**. The 5X GTAC cis-elements and the mutated GTACm version were inserted into the pHIS2 vector (Yu et al., 2017) and used to co-transform the Y2HGold cells. The transformants were streaked onto SD/-Trp and SD/-Trp/-His/-Ade media and incubated for 3–5 days at 30°C.

### 2.5 Generation of transgenic *Arabidopsis* plants overexpressing *ZmSBP17*

The pCAMBIA2300-*ZmSBP17*-GFP vector was introduced into the *Agrobacterium tumefaciens* strain EHA105, which was then used to transform *A. thaliana* via the floral dip method (Clough and Bent, 1998). Transgenic seeds (T_0_) were selected on MS inorganic salts (Murashige and Skoog, 1962) medium supplemented with 50 mg·L^−1^ kanamycin. At the 7–8 leaf seedling stage, the lowest leaves were used to identify PCR-positive plants using detection primers (**Supplementary File 1**). Homozygous lines were selected on MS medium containing 50 mg·L^−1^ kanamycin.

### 2.6 Osmotic treatment of transgenic *Arabidopsis* plants and phenotype analysis

For osmotic stress seed germination assays, WT and T_3_ homozygous OE seeds were sterilized and vernalized at 4°C for 3 days. They were then sown on half-strength MS medium supplemented with either 100 mM NaCl or 8% PEG. Germination rates were assessed after 7 days. All germination assays were performed in triplicate. To evaluate the sensitivity of adult plants to osmotic stress, 4-week-old WT and OE plants were irrigated with either 200 mM NaCl or 10% PEG at 3-day intervals for 7 days and subsequently photographed.

### 2.7 Histochemical analysis under osmotic treatment

For histochemical analysis, 4-week-old WT and OE plants were treated with 200 mM NaCl or 10% PEG for 36 h. The entire plants were then used for staining. DAB and NBT staining were performed as described by Zhang et al. (2011) with slight modifications.

### 2.8 Measurements of enzyme activity in transgenic *Arabidopsis*

For detecting the activity of ROS-scavenging enzymes, 4-week-old WT and OE plants were irrigated with 200 mM NaCl or 10% PEG for 36 h, and then rosette leaves were collected. The activities of peroxidase (POD), superoxide dismutase (SOD), and catalase (CAT) were measured as described by Wang et al. (2019). Ascorbate peroxidase (APX) was extracted as described by Erinle et al. (2018) with slight modifications.

## 3 Results

### 3.1 Analysis of *ZmSBP17*

**Figure 1:**
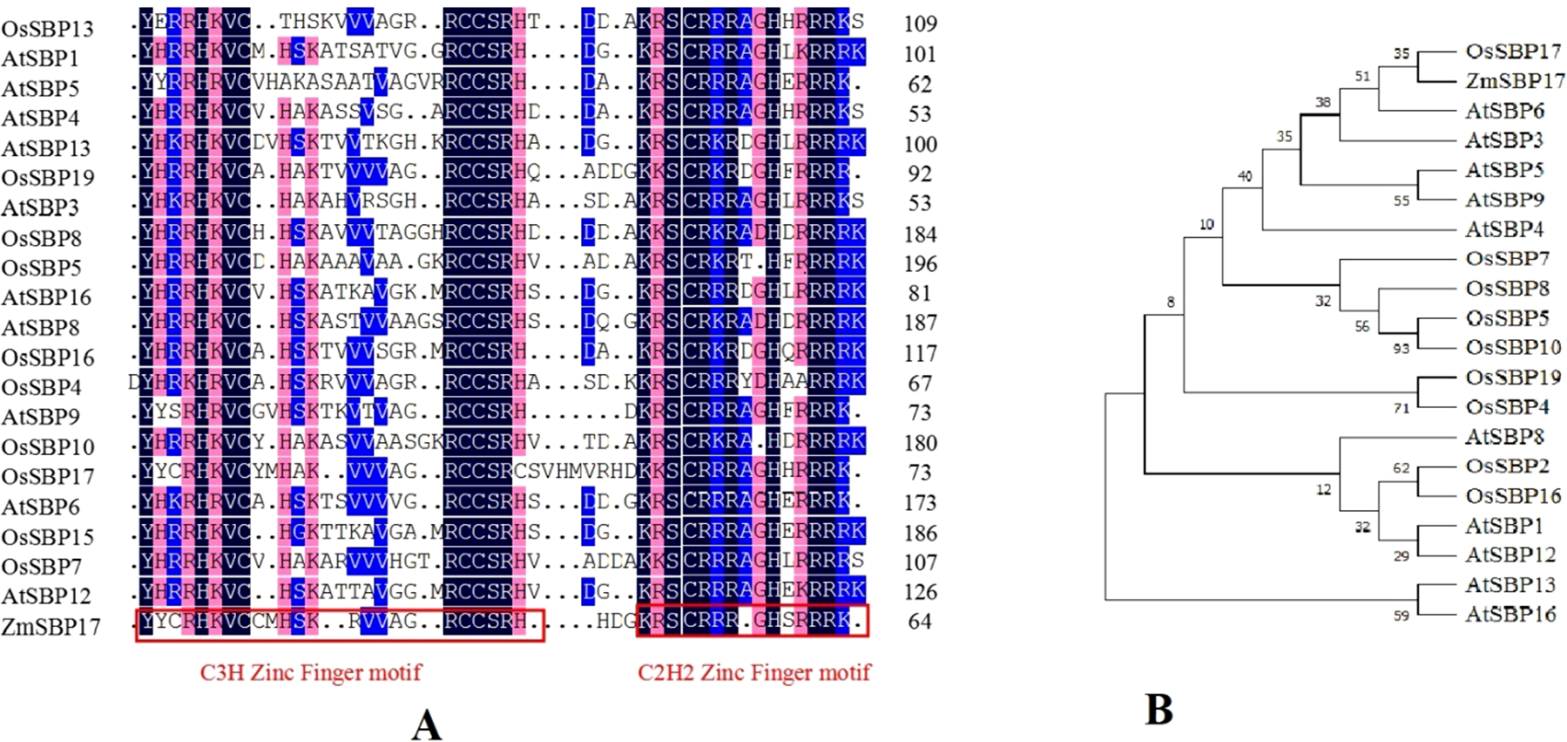
Multiple alignments and phylogenetic relationships of ZmSBP17 with orthologs in rice and *Arabidopsis*. **(A)** Multiple alignments of ZmSBP17 with homologous proteins in rice and *Arabidopsis*. Different background colors display similar acid sequences. Red boxes indicate conserved C3H and C2H2 zinc finger motifs, respectively. **(B)** Phylogenetic relationships of ZmSBP17 with orthologs in rice and *Arabidopsis*.

Following the expression analysis of *SBP*s under salt stress by qRT-PCR in our previous study, a putative salt stress-related *SBP* from the Hei maize inbred line was isolated and named *ZmSBP17* (GenBank accession no. NM_001308478) according to blastp results (**Supplementary File 2**). The open reading frame of ZmSBP17 is 1137 bp long, encoding a 379 amino acid polypeptide. Amino acid sequence alignment and phylogenetic analysis of ZmSBP17 homologs are shown in **Figure 1**. The average identity homology of ZmSBP17 with those in rice and *Arabidopsis* was 12.9%. Multiple sequence alignment by DNAMAN revealed it to have C2HC and C3H domains at the N terminus (**Supplementary File 3**). Phylogenetic analysis showed that ZmSBP17 was relatively close to OsSBP17, but ZmSBP17 was less identical to homologs other than OsSBP17, which suggested that ZmSBP17 markedly differs from other members. Analysis of the upstream 2000 bp promoter region of ZmSBP17 revealed the presence of multiple elements related to abiotic stress, including abscisic acid response element (ABRE), antioxidant response element (ARE), long terminal repeat (LTR), and MYB-binding sites (MBS) (**Supplementary File 4**).

### 3.2 ZmSBP17 expression and protein localization

**Figure 2:**
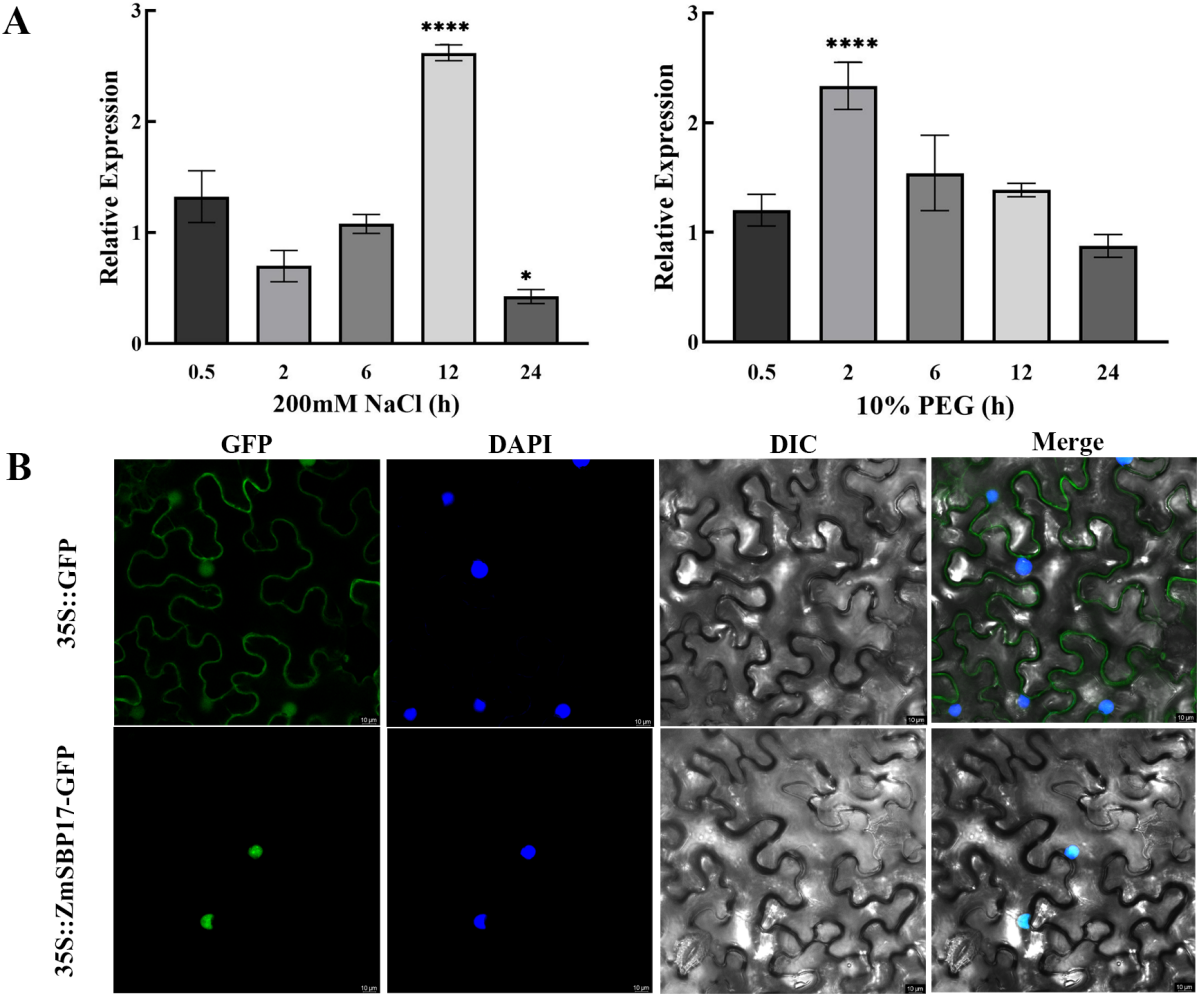
*ZmSBP17* expression and subcellular localization. **(A)** Expression patterns of *ZmSBP17* under different treatments. Expression pattern of *ZmSBP17* in leaves. Relative expression levels were normalized to 1 in Hei plants without stress (0 h). Values are presented as mean (± SD) of three biological replicates. * and **** significantly higher values in treatment samples than in control plants (0 h) at *p* ≤ 0.05 and *p* ≤ 0.0001 as determined using Student’s *t*-test, respectively. **(B)** Subcellular localization of ZmSBP17 in the tobacco epidermal cells. GFP, green fluorescence protein images; DAPI, 4’, 6-diamidino-2-phenylindole (DAPI) stained images; DIC, brightfield images; Merged, merged brightfield and GFP images. Scale bar = 10 μm.

The expression pattern of *ZmSBP17* under abiotic stress was investigated by subjecting the three-leaf stage maize seedlings to the indicated treatment. *ZmSBP17* expression increased significantly and reached a peak at 12 and 2 h under salt and PEG stresses, respectively, indicating that it may participate in the osmotic stress responses in maize (**Figure 2A**; **Supplementary File 5**).

The subcellular localization of *Z*mSBP17 constructed transient expression vector pCAMBIA2300-*ZmSBP17*-GFP (**Supplementary File 2**) and pCAMBIA2300- GFP vectors were transformed into the tobacco epidermal cells. The green fluorescence signals of expressing GFP gene was monitored by a confocal laser scanning microscope. As shown in **Figure 2B**, the pCAMBIA2300-ZmSBP17-GFP fusion protein was specifically detected in the nucleus compare to the control distributed throughout the cell.

### 3.3 Transcriptional activity assay of ZmSBP17

**Figure 3:**
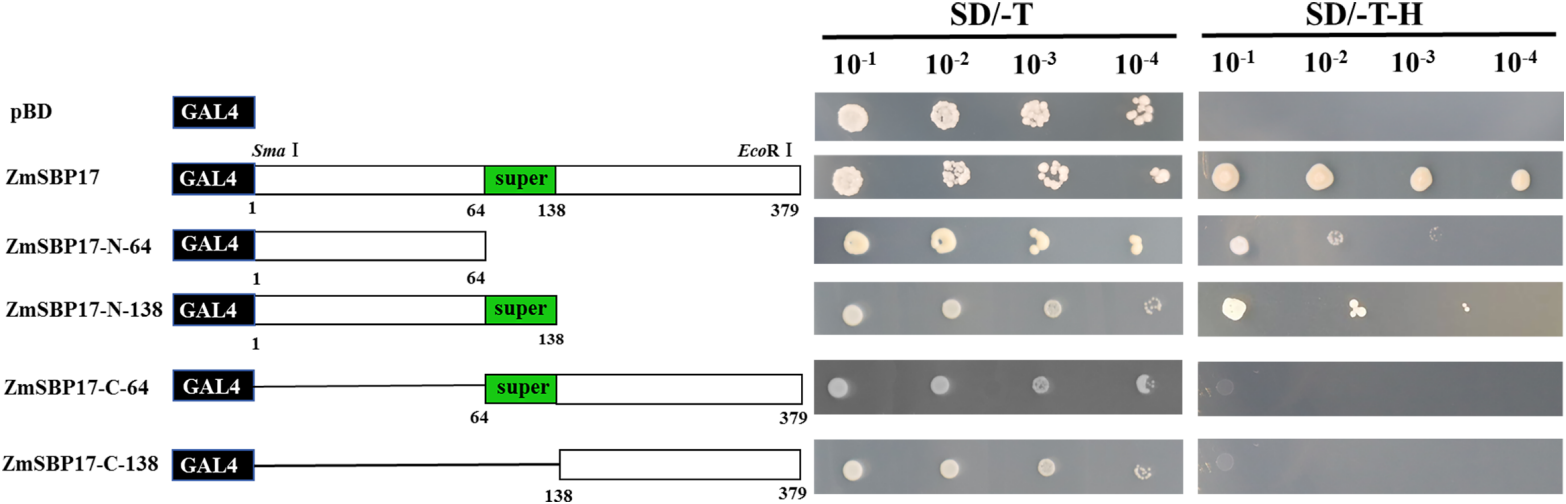
Analysis of the transcriptional activation capacity of the full-length or partially deleted ZmSBP17 in yeast. Numbers indicate amino acid positions. Transformants carrying the empty pGBKT7 plasmid were used as a negative control. Transformants were examined on SD/-Trp (growth control) and SD/-Trp/-His media.

The full-length *ZmSBP17* was fused to the GAL4 (GAL4-BD) binding domain of pGBKT7 to confirm if ZmSBP17 has transcription activation ability. The constructed pGBKT7-*ZmSBP17* vector is shown in **Supplementary File 3**. A comparison of the growth phenotype of the pGBKT7-*ZmSBP17* and pGBKT7 recombinant yeast revealed that they can survive on SD/-Trp selective medium with the same colony size, indicating the successful transfer of each vector into the Y2H cells. However, only those containing the pGBKT7-*ZmSBP17* vector grew normally on SD/-Trp-His selective medium, suggesting that ZmSBP17 was able to activate the *HIS* reporter gene for survival. Thus, ZmSBP17 acts as a transcriptional activator in yeast (**Figure 3**).

Protein sequence analysis revealed that the SBP superfamily domains are present in the ZmSBP17 protein (from 64 to 138 amino acid residues). To determine which domain in ZmSBP17 was the transcriptional activator, four deletion fragments were inserted into pGBKT7 and used to transform Y2H cells. All transformants grew well on the SD/-Trp medium. Contrary to those carrying the N-64 and N-138 fragments, the yeast cells carrying the C-64 fragment of ZmSBP17 could not grow on the selection medium. Meanwhile, yeast cells carrying the N-138 SBP domain fragment (amino acids 64–138) grew better than those carrying the N-64 fragment on the selection medium. These results indicate that the SBP domain enhanced the transcription activation ability of ZmSBP17, and the 1–64 amino acid fragment was crucial for this ability.

### 3.4 ZmSBP17 positively regulates osmotic responses in plants

**Figure 4:**
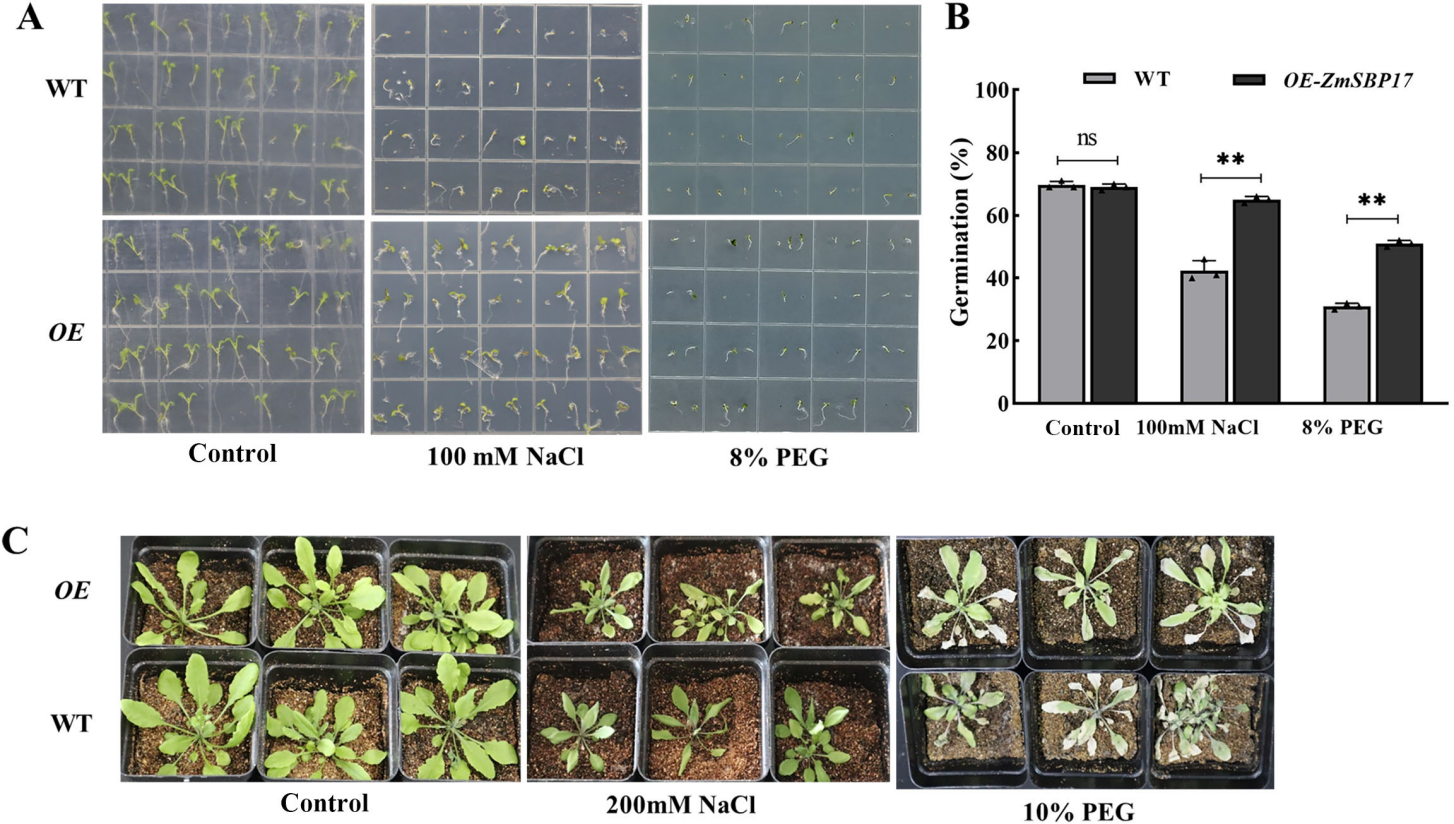
*ZmSBP17* positively regulates osmotic stress response. **(A)** Germination assay. Seeds of the OE and WT plants were cultivated for 7 days on a half-strength MS medium supplemented with 100 mM NaCl and 5% PEG. **(B)** Statistical analysis of the germination rates; 72 seeds were used for each experiment. **P < 0.01 as determined by Student’s *t*-test. ns, no significance. **(C)** Phenotypic assay of soil-grown plants; 4-week-old OE and WT plants were treated with 200 mM NaCl and 10% PEG for 7 days.

pCAMBIA2300-*ZmSBP17* was used to transform the WT *A*. *thaliana* Columbia-0 plants to characterize whether *ZmSBP17* is associated with the osmotic stress response. The transgenic plants were screened on kanamycin and identified by PCR (**Supplementary Figure 4**). The T_3_ plants were used for further analysis. No significant differences in seed germination rate were observed between transgenic and WT plants on MS medium. However, in the presence of 100 mM NaCl and 8% PEG, an obvious reduction in germination rate was detected in both transgenic and WT seeds but was significantly higher in the transgenic than WT seeds (**Figures 4A, B**; **Supplementary File 6**).

For further functional analysis of *ZmSBP17*, the adult stage *ZmSBP17-*OE lines were examined in soil. Under normal conditions, no significant phenotypic variations between the OE lines and WT were observed. However, post-treatment with 200 mM NaCl and 10% PEG for 36 h, the stems and leaves of the WT showed obvious wilting, with some leaves turning yellow and drying. However, the wilting of transgenic *A. thaliana* was significantly less severe than that of the WT under PEG stress. The stems and leaves of the transgenic plants remained relatively upright, and leaf yellowing was also less pronounced compared with the WT. However, under salt stress, there was no apparent difference between the WT and transgenic plants (**Figure 4C**). These results indicate that ZmSBP17 may improve the tolerance of *Arabidopsis* seeds at various stages to PEG.

### 3.5 ZmSBP17 positively regulates ROS-scavenging systems under osmotic stress

**Figure 5:**
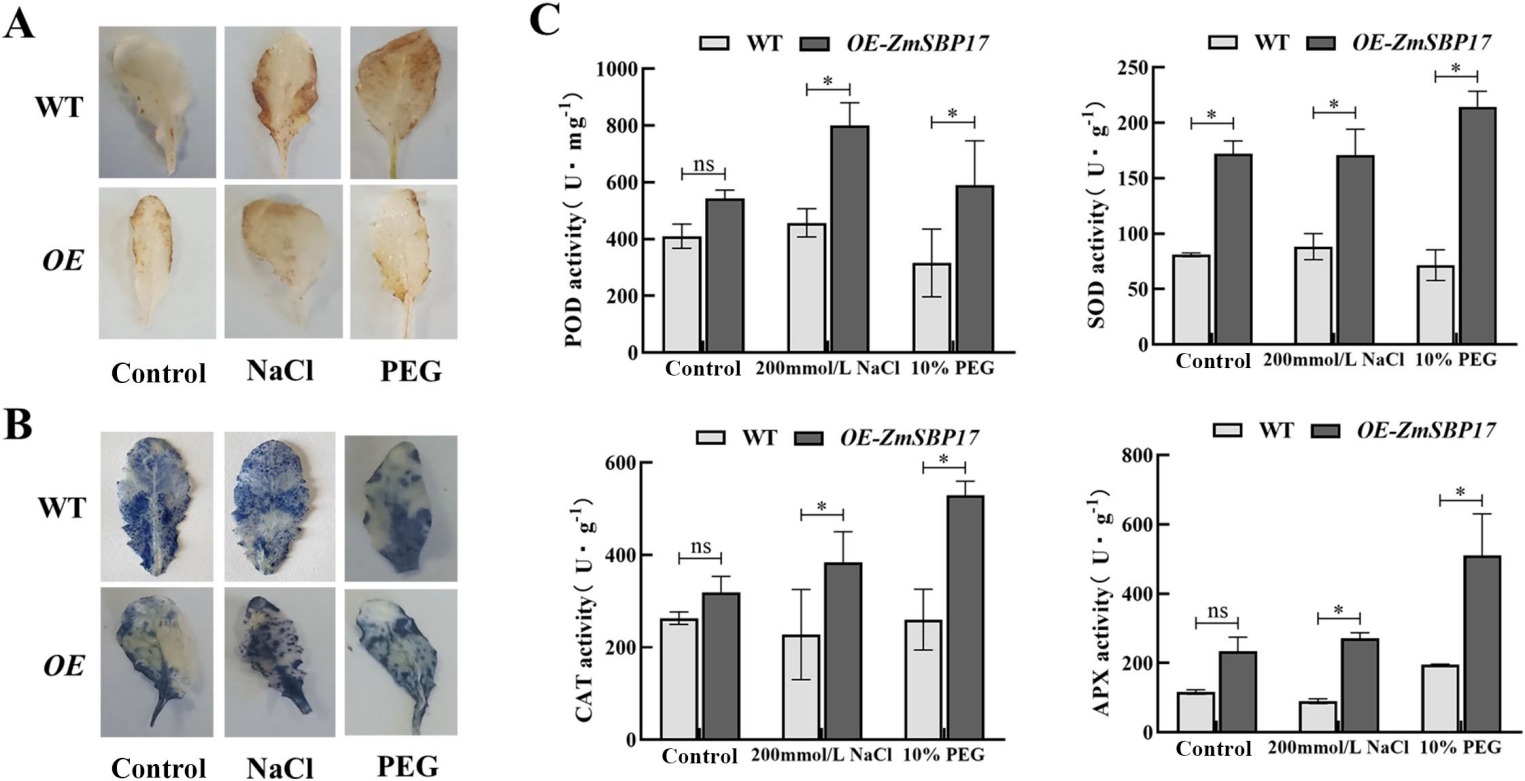
Detection of ROS accumulation and scavenging capability. **(A, B)** Analysis of H_2_O_2_ and O_2_^-^ accumulation using DAB and NBT staining, respectively. **(C)** Measurement of POD, SOD, CAT, and APX activities. Values are presented as the mean (± SD) of three biological replicates. Upper or lower whiskers delineate maximum and minimum values, respectively. *P < 0.05 as determined by Student’s *t*-test. ns, no significance.

The levels of H_2_O_2_ and O_2_^¯^ that are key ROS species were detected in *ZmSBP17* OE line and WT plants using DAB and NBT staining, respectively to investigate whether *ZmSBP17* is associated with the regulation of ROS scavenging under osmotic stress. After treatment with 200 mM NaCl and 10% PEG, the staining in the leaf tissues of the WT and OE lines increased significantly. However, the DAB staining was deeper in the WT than in the OE lines, and there was no significant difference in NBT intensity between the WT and OE lines under PEG stress. Under salt stress, the DAB and NBT staining was deeper in the WT than in the OE line (**Figures 5A, B**). Moreover, the activities of key ROS-scavenging enzymes POD, SOD, CAT, and APX were assessed in the OE and WT plants at 0 and 36 h after osmotic treatment. As shown in **Figure 5C**, the activities of all four increased significantly in the OE line compared to WT plants after stress treatment (**Supplementary File 6**). These results indicate that ZmSBP17 might play a role in modulating the ROS-scavenging system by increasing the activities of protective enzymes under osmotic stress.

### 3.6 ZmSBP17 positively regulates osmotic stress-related gene expression in transgenic plants

**Figure 6:**
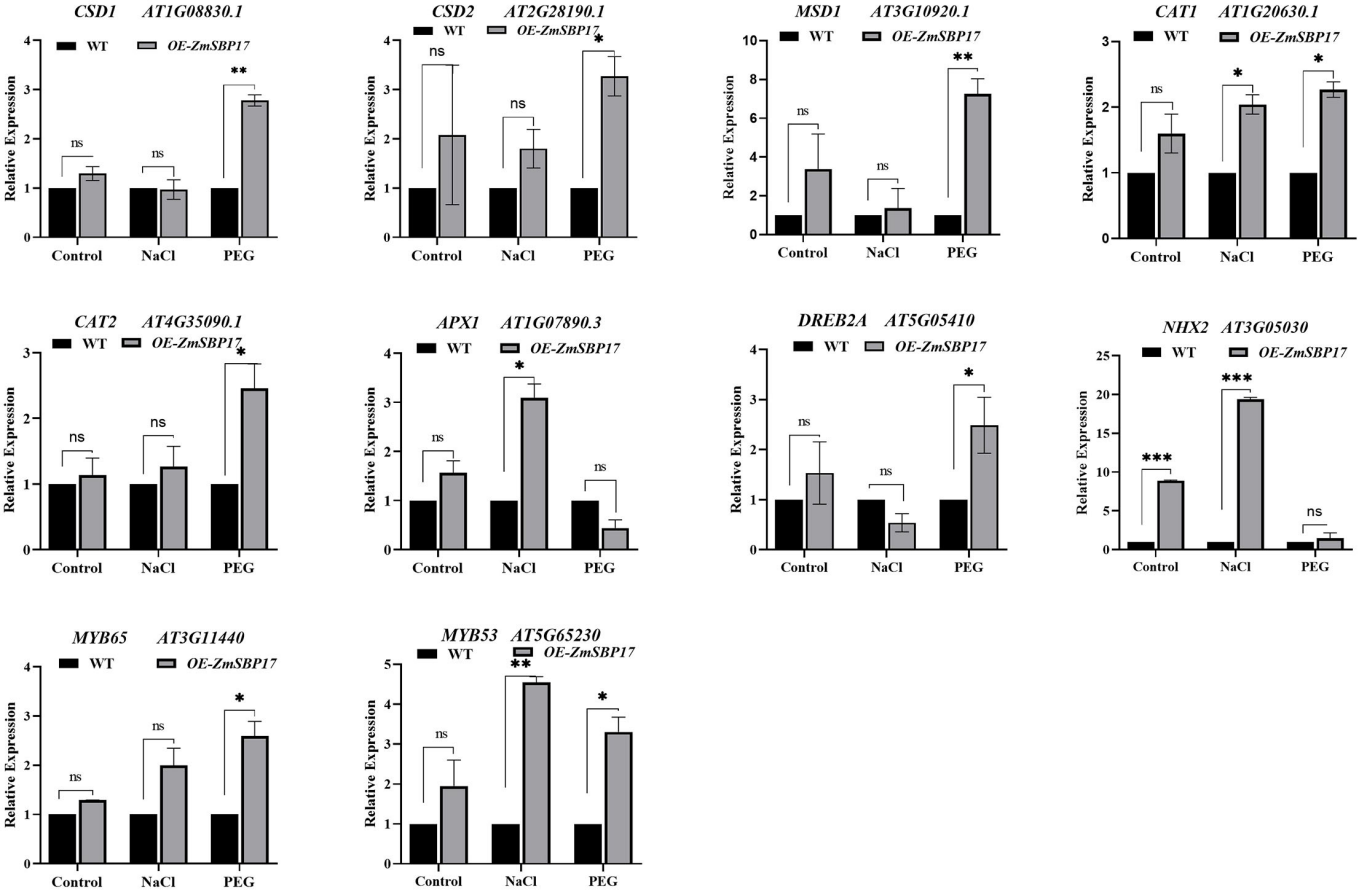
Relative expression levels of abiotic stress-responsive genes in OE and WT plants under salt and PEG stress. Expression of each gene in the WT was set to 1. Values are presented as the mean (± SD) of three biological replicates. *, ** and *** indicate significantly higher values in the treatment samples than in the WT at *p* ≤ 0.05, *p* ≤ 0.01 and *p* ≤ 0.001, respectively, determined using the Student’s *t*-test. ns, no significance.

The expression levels of the abiotic stress-responsive genes—*CSD1*, *AT1G08830.1*; *CSD2*, *AT2G28190.1*; *MSD1*, *AT3G10920.1*; *CAT1*, *AT1G20630.1*; *CAT2*, *AT4G35090.1*; *APX1*, *AT1G07890.3*; *DREB2A*, *AT5G05410*; *NHX2*, *AAT3G05030*; *MYB65*, *AT3G1140*; and *MYB53*, *AT5G65230*—were analyzed in WT and transgenic plants by real-time qRT-PCR to better understand the mechanistic basis of the improved osmotic stress tolerance exhibited by the *ZmSBP17*-transgenic plants. As shown in **Figure 6**, the expression levels of 10 genes increased significantly in transgenic plants compared to WT under salt or PEG stresses. For example, the expression levels of the genes encoding the ROS-scavenging enzymes, *SOD*s (*CSD1/2* and *MSD1*) and *CAT*s (*CAT1/2*), were significantly elevated in the *ZmSBP17*-OE lines than that in the WT under PEG stress, while the expression levels of *CAT1* and *APX1* were significantly higher under salt stress. Moreover, the expression level of the drought-related gene (*DREB2A*) was significantly more than in WT under PEG stress. The expression levels of the Na^+^ or K^+^ transport-related gene (*NHX1*) were not only significantly lower in the WT than in transgenic plants under normal conditions but also significantly lower in the WT than in the transgenic plants after salt stress to maintain Na^+^ and K^+^ balance. Additionally, the expression level of the MYB TF family genes (*MYB53/65*) in the OE and WT plants was assessed. The expression level of *MYB65* was significantly higher in the transgenic plants than in WT after PEG stress, while that of *MYB53* was significantly enhanced in the transgenic plants than that in the WT after salt and PEG stress.

These results indicate that the overexpression of *ZmSBP17* may aid in improving osmotic stress tolerance in plants by elevating the expression of ROS-scavenging enzymes encoding, drought-related, Na^+^ or K^+^ transport-related, and MYB TF family genes.

### 3.7 ZmSBP17 binds to the GTAC ([G/T] [A/G/T]) core sequence

**Figure 7:**
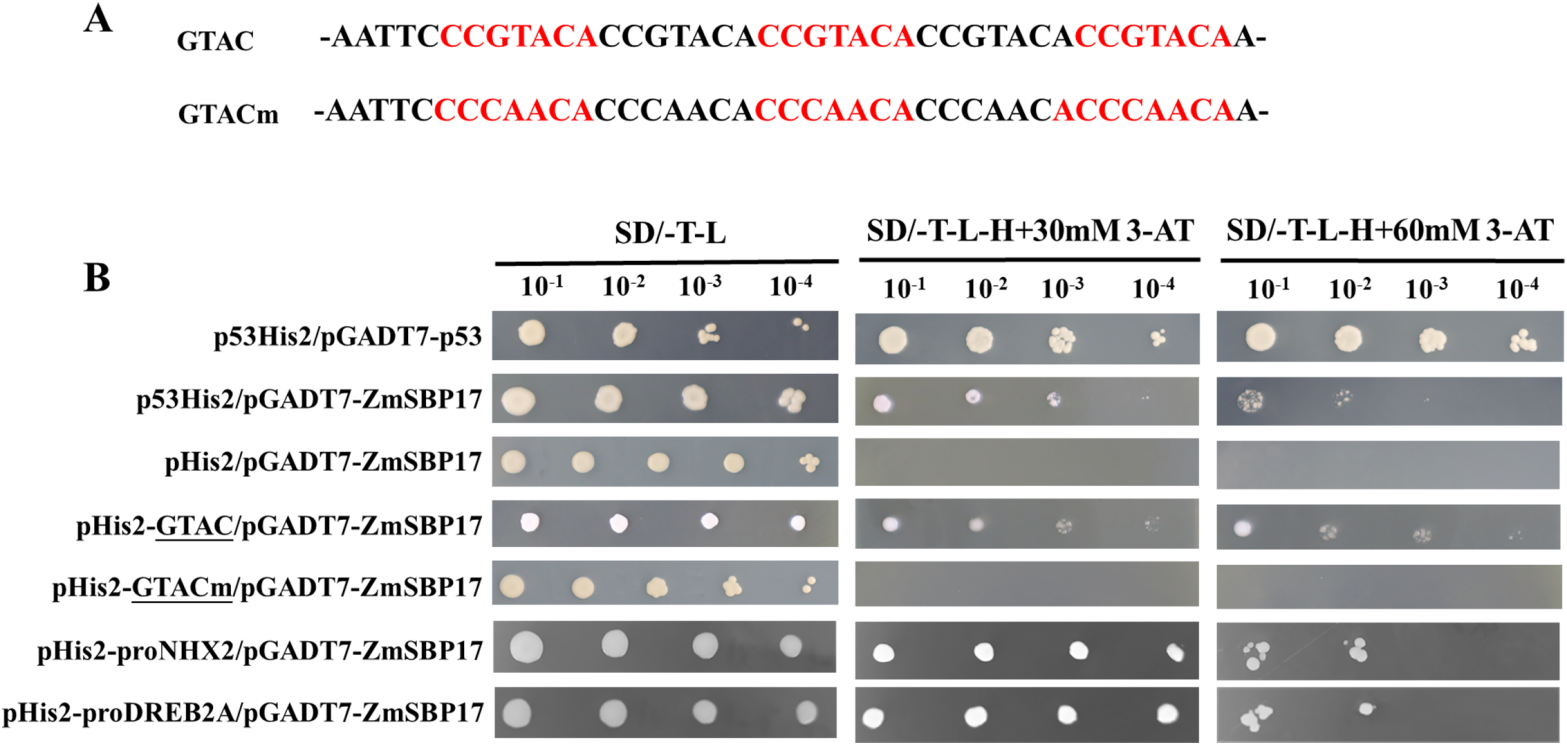
ZmSBP17 binds to GTAC core sequences and the promoters of target genes. **(A)** Sequences of the WT and mutated GTAC cis-elements; **(B)** Yeast one-hybrid assays displaying the binding ability of ZmSBP17 to the GTAC cis-element in the promoters of the target genes; yeast co-transformants with the effector vector and each reporter were analyzed by culturing serial dilutions (10^−1^, 10^−2^, 10^−3^, and 10^−4^) on a selection medium containing 3-AT. p53His2/pGADT7-p53: positive controls, pHis2/pGADT7-ZmSBP17: negative controls.

SBP TFs could bind to the GTAC-box ([C][C]GTAC[A/G]) cis-elements. Therefore, *ZmSBP17* was cloned into pGADT7 while the GTAC core sequence and mutated elements were repeated five times and cloned separately into pHIS2. Growth assays revealed that the yeast co-transformed with GTAC and *ZmSBP17* can grow better on the selection medium, while those containing the GTACm sequence displayed the same growth as the negative control pHis2 (**Figure 7B**). These results indicate that the core GTAC-box sequence is crucial for the ZmSBP17 protein to recognize its target genes.

The promoters (from −1500 to −1) of 10 putative target genes that were significantly upregulated by ZmSBP17 were analyzed to investigate the distribution of the core GTAC-box sequences. The promoters of all putative target genes contained more than four GTAC boxes. The schematic diagrams of the promoters of these genes are shown in **Supplementary File 7**. The GTAC-box may be essential for ZmSBP17 to regulate its target genes.

The direct binding of ZmSBP17 to the promoters of the putative target genes containing GTAC-box core sites was verified. The promoter fragments of *NHX2* and *DREB2A* containing the core GTAC-box sequences were isolated through genomic PCR and inserted into pHIS2 in yeast to identify the binding affinity to ZmSBP17 on the selection medium. The results show that all co-transformants exhibited binding affinity on the selection medium (**Figure 7B**), indicating that ZmSBP17 can directly interact with the *NHX1* and *DREB2A* promoter fragments containing the core GTAC-box sequences.

## 4 Discussion

TFs can act as activators or repressors to regulate the expression of various downstream genes, thereby enhancing stress tolerance in plants (He et al., 2019; Sun et al., 2022). The SBP-box gene family, a group of plant-specific TFs, plays a significant role in the growth and development of many plants (Shao et al., 2019; Tong et al., 2020; Li et al., 2021). However, the activation mechanism of SBP-box genes related to abiotic stress in maize remains unexplored. In this study, an SBP TF, ZmSBP17, was isolated from maize based on the salt stress transcriptome database (**Supplementary File 2**). The SBP domains of ZmSBP17 were found to be highly similar, with a conserved sequence at specific positions, a nuclear localization signal, and two zinc finger domains (Klein et al., 1996; Yamasaki et al., 2006). This study confirmed that ZmSBP17 contains two zinc finger domains, similar to those in other plants (**Figure 1**). Additionally, it was demonstrated that ZmSBP17 possesses transcriptional activation ability, with the N-terminal region exhibiting stronger activity than the C-terminal region (**Figure 3**).

Moreover, the expression of *ZmSBP17* was significantly induced by salt and PEG stress during the seedling stage of maize (**Figure 2A**). This induction may be attributed to the presence of cis-elements such as ABRE, ARE, LTR, and MBS in the *ZmSBP17* promoter, which are associated with abiotic stress responses. In *Arabidopsis*, MBS cis-elements in the gene promoter are involved in responses to drought, salt, and low-temperature stress (Yao et al., 2022; Li et al., 2023). Similarly, the ABRE element in the *Arabidopsis* RD29A promoter plays a crucial role in abiotic stress responses by driving and regulating the expression of stress-related TFs AREB1 and AREB2 (Bihmidine et al., 2013). Therefore, this study focuses on elucidating the molecular mechanism of *ZmSBP17* in response to drought and salt stress.

Numerous studies have shown that abiotic stress can lead to excessive accumulation of ROS in plant cells, resulting in cellular oxidative stress. This condition can limit growth, development, and metabolism, ultimately leading to cell death (Singh et al., 2018). However, plants have evolved complex defense mechanisms to counteract stress-induced damage, including antioxidant enzyme systems such as SOD, POD, CAT, and APX (Gelaw and Sanan-Mishra, 2024; Kiratli et al., 2024). For instance, overexpression of *SbNAC2* from Sorghum in *Arabidopsis* and rice enhances antioxidant enzyme activities and induces stress-responsive genes, thereby improving tolerance to abiotic stress (Jin et al., 2021). Similarly, overexpression of *NtWRKY65* in tobacco plants increases salinity tolerance by elevating antioxidant enzyme activities compared with WT plants (Zhang et al., 2024). In this study, under salt and PEG stress, overexpression of *ZmSBP17* in *Arabidopsis* significantly improved the germination rate compared with the WT (**Figure 4**). At the seedling stage, the activities of SOD, POD, CAT, and APX in transgenic *Arabidopsis* plants were all significantly higher than those observed in WT plants (**Figure 5**).

TFs regulate gene expression by binding to cis-acting elements in the promoters of abiotic stress-related genes. For instance, SPL1, SPL3, SPL7, and SPL8 in *Arabidopsis* can bind to the AP1 and FUL promoters, which contain the GTAC core sequence (Cardon et al., 1997; Birkenbihl et al., 2005; Kropat et al., 2005; Shikata et al., 2009). Our study also revealed that *ZmSBP17* specifically binds to the GTAC core sequence (**Figure 7**), suggesting that SBPs from different species can recognize similar core sequences to regulate biological functions. Additionally, we discovered that SBP-specific GTAC core sequences are widely present in the promoters of stress-related genes, including FSD3, CBF3, DREB1A/19, NHX1/2/6, and MYB53/65/99 (**Supplementary File 7**). Notably, DREB2A, NHX2, and MYB53/65 were significantly upregulated in *ZmSBP17*-OE plants under osmotic stress (**Figure 6**). DREB2A regulates abiotic stress responses by activating downstream genes (Agarwal et al., 2010; Sato et al., 2014; Chen et al., 2016). NHX2, a vacuolar Na^+^/H^+^ antiporter, enhances tolerance to drought and salt stresses by reducing Na^+^ content in leaves, showing improved root health under salt stress, and retaining more water during drought (Wang et al., 2016). High expression of *NHX1* in Arabidopsis can enhance salt tolerance in saline–alkaline environments (Pérez-Martín et al., 2022; Eduardo et al., 2007). Additionally, several MYB family members, such as AtMYB41, AtMYB44, AtMYB88, and AtMYB102, positively contribute to *Arabidopsis*’s response to abiotic stress (Denekamp and Smeekens, 2003; Cominelli et al., 2008; Jung et al., 2008; Xie et al., 2010). Transgenic *Arabidopsis* and potato plants overexpressing a mutated version of MYB33/65/101 with altered miR159 recognition sites displayed hypersensitivity to ABA and relatively high tolerance to drought (Xue et al., 2017). Collectively, these results indicate that the overexpression of *ZmSBP17* in *Arabidopsis* may enhance osmotic stress tolerance by upregulating stress-responsive genes, supporting *ZmSBP17* as a positive regulator. However, further investigations are needed to identify the cis-elements and downstream genes targeted by *ZmSBP17* in maize.

## 5 Conclusion

**Figure 8:**
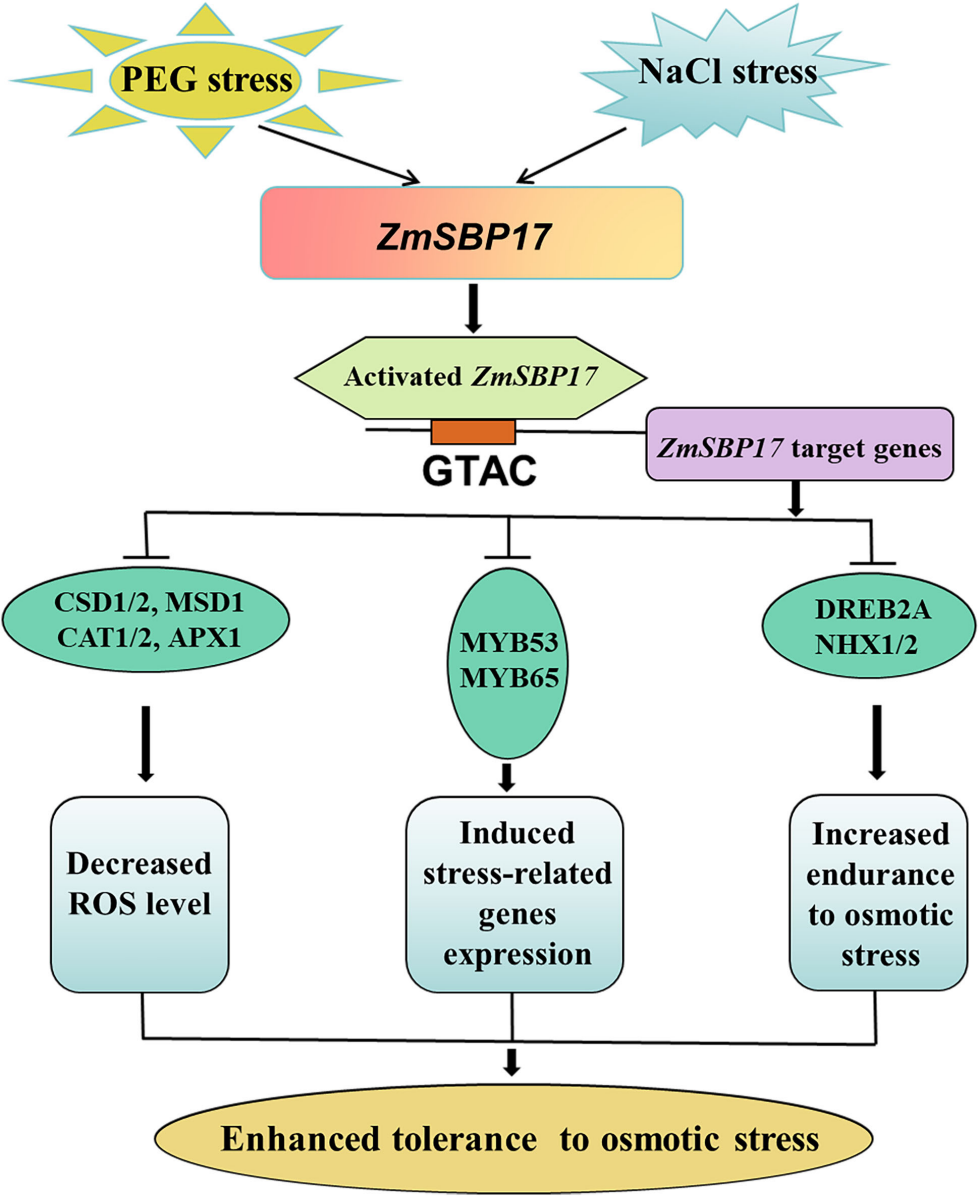
Working model of ZmSBP17 under osmotic stress. Salt or PEG stress induces the expression of ZmSBP17. Activated ZmSBP17 enhances stress-associated genes expression by binding to GTAC core sequences, which results in increasing ROS scavenging capability, induced stress-related genes expression, and increased endurance to osmotic stress. These physiological changes finally enhanced the tolerance of ZmSBP17 transgenic plants to osmotic stress.

In conclusion, we demonstrated that the expression of *ZmSBP17* in the inbred line Hei maize was induced by salt and PEG stress. *ZmSBP17* was confirmed to be a transcriptional activator and was able to bind to the GTAC core sequences to downregulate target genes. Overexpression of *ZmSBP17* in *Arabidopsis* significantly increased tolerance to osmotic stress during the germination and seedling stages. This enhanced tolerance to abiotic stress was likely caused by a decrease in ROS levels through improved ROS clearance ability. Another possible factor may be the altered expression of a series of stress-related genes in ZmSBP17-OE plants (**Figure 8**). These strategies may collectively provide a more robust defense against osmotic stress.

## Data Availability

The datasets presented in this study can be found in online repositories. The names of the repository/repositories and accession number(s) can be found in the article/**Supplementary Material**.

## Supplementary material

Supplementary File 1 Primers used for qRT-PCR.

Supplementary File 2 Expression levels of *SBP*s in maize under salt stress and sequence analysis of *ZmSBP17*.

Supplementary File 3 Identification of the recombinant plasmid pGBKT7-*ZmSBP17*.

Supplementary File 4 Cis-elements in the ZmSBP17 promoter related to abiotic stress.

Supplementary File 5 Multiple sequence alignment of the SBP family.

Supplementary File 6 qRT-PCR analysis of *ZmSBP17* in maize plants under salt and PEG stress.

Supplementary File 7 Overexpression of *ZmSBP17* in *Arabidopsis thaliana*.

Supplementary File 8 Germination rates of OE and WT seeds under salt or PEG stress and activities of ROS-scavenging enzymes.

Supplementary File 9 Distribution of the GTAC-box core sequences in the promoters of genes regulated by ZmSBP17.
