# Supplementary material for: The maize gene ZmSBP17 encoding an SBP transcription factor confers osmotic resistance in transgenic Arabidopsis
Source: Front Plant Sci. 2024 Nov 7;15:1483486. doi: 10.3389/fpls.2024.1483486 (PMC11578699; doi:10.3389/fpls.2024.1483486)
Supplement: Supplementary File 2 — Expression levels of SBPs in maize under salt stress and sequence analysis of ZmSBP17. [file Table2.docx]

>ZmSBP17

MATGGGSSRSDDVRGLKFGKKIYFEQDGGSGSGAGAVGGRKGKGVATGGARPASAASAAQPPRCQVDGCG

VDLSAVKQYYCRHKVCNMHSKEPRVFVAGIEQRFCQQCSRFHQLHEFDQGKRSCRRRLIGHNERRRKPPP

GPLTSRYGRLAASLQEPGRFRSFLLDFSYPRVPSSVRDAWPGIQHGGDRMLGTVQWHGHQEPPHPHRSAA

AGYGNHAAYNCHGGLVAGGAPMLSSAAFELPPGGCVAGVAADSSCALSLLSTQPWDTTSHDHRSPAMPAA

GAFDGTPVAPSVMASSYAASSAWTGSRDPAADGARNAQRLDDALHLVHPGSAAVHFSGELELALQGSGGP

PHLPRVDHGGSGGGTFNHSTTSAMNWSL

>OsSBP13

MDRKDKARKNFSSSSSSSAASMAALAAAAAAGDGGAALPSPMEEDKKPRLVASSLAPVAGGGGGGSSSSA

AVAAGASSSSSSSSVAAAARRGAGRAGGGAPSGGGGGPRCQVERCGVDLSEAGRYNRRHKVCQTHSKEPV

VLVAGLRQRFCQQCSRFHELTEFDDAKRSCRRRLAGHNERRRKSAADTAHGENCRHADQDAGRSHQGTGN

PPFQIR

>AtSBP1

MEARIDEGGEAQQFYGSVGKRSVEWDLNDWKWDGDLFLATQTTRGRQFFPLGNSSNSSSSCSDEGNDKKR

RAVAIQGDTNGALTLNLNGESDGLFPAKKTKSGAVCQVENCEADLSKVKDYHRRHKVCEMHSKATSATVG

GILQRFCQQCSRFHLLQEFDEGKRSCRRRLAGHNKRRRKTNPEPGANGNPSDDHSSNYLLITLLKILSNM

HNHTGDQDLMSHLLKSLVSHAGEQLGKNLVELLLQGGGSQGSLNIGNSALLGIEQAPQEELKQFSARQDG

TATENRSEKQVKMNDFDLNDIYIDSDDTDVERSPPPTNPATSSLDYPSWIHQSSPPQTSRNSDSASDQSP

SSSSEDAQMRTGRIVFKLFGKEPNEFPIVLRGQILDWLSHSPTDMESYIRPGCIVLTIYLRQAETAWEEL

SDDLGFSLGKLLDLSDDPLWTTGWIYVRVQNQLAFVYNGQVVVDTSLSLKSRDYSHIISVKPLAIAATEK

AQFTVKGMNLRQRGTRLLCSVEGKYLIQETTHDSTTREDDDFKDNSEIVECVNFSCDMPILSGRGFMEIE

DQGLSSSFFPFLVVEDDDVCSEIRILETTLEFTGTDSAKQAMDFIHEIGWLLHRSKLGESDPNPGVFPLI

RFQWLIEFSMDREWCAVIRKLLNMFFDGAVGEFSSSSNATLSELCLLHRAVRKNSKPMVEMLLRYIPKQQ

RNSLFRPDAAGPAGLTPLHIAAGKDGSEDVLDALTEDPAMVGIEAWKTCRDSTGFTPEDYARLRGHFSYI

HLIQRKINKKSTTEDHVVVNIPVSFSDREQKEPKSGPMASALEITQIPCKLCDHKLVYGTTRRSVAYRPA

MLSMVAIAAVCVCVALLFKSCPEVLYVFQPFRWELLDYGTS

>AtSBP5

MEGQRTQRRGYLKDKATVSNLVEEEMENGMDGEEEDGGDEDKRKKVMERVRGPSTDRVPSRLCQVDRCTV

NLTEAKQYYRRHRVCEVHAKASAATVAGVRQRFCQQCSRFHELPEFDEAKRSCRRRLAGHNERRRKISGD

SFGEGSGRRGFSGQLIQTQERNRVDRKLPMTNSSFKRPQIR

>AtSBP4

MEGKRSQGQGYMKKKSYLVEEDMETDTDEEEEVGRDRVRGSRGSINRGGSLRLCQVDRCTADMKEAKLYH

RRHKVCEVHAKASSVFLSGLNQRFCQQCSRFHDLQEFDEAKRSCRRRLAGHNERRRKSSGESTYGEGSGR

RGINGQVVMQNQERSRVEMTLPMPNSSFKRPQIR

>AtSBP13

MDWNFKLSSGYLSGFDQEPDLSPMDGSISFGGSSQSKADFSFDLKLGRNIGNSSSVFGDTEQVISLSKWK

DSALAKPEGSRSSSSKRTRGNGVGTNQMPICLVDGCDSDFSNCREYHKRHKVCDVHSKTPVVTINGHKQR

FCQQCSRFHALEEFDEGKRSCRKRLDGHNRRRRKPQPEHIGRPANFFTGFQGSKLLEFSGGSHVFPTTSV

LNPSWGNSLVSVAVAANGSSYGQSQSYVVGSSPAKTGIMFPISSSPNSTRSIAKQFPFLQEEESSRTASL

CERMTSCIHDSDCALSLLSSSSSSVPHLLQPPLSLSQEAVETVFYGSGLFENASAVSDGSVISGNEAVRL

PQTFPFHWE

>OsSBP19

MEWAAAATKAASWGMAVAAAAAADDAGPTMLSFAGPSSSSSSPDAAAAAAAAAAAALHDFSVRARPAAAAPATRRARGGSGGGGGGGGGAEACSVDGCRSDLSRCRDYHRRHKVCEAHAKTPVVVVAGQEQRFCQQCSRFHNLAEFDDGKKSCRKRLDGHNRRRRKPQHDALNPRSFLPYHQANQFSVYPQTFPIADQNADALMRPLDRH

PPFSISFSGTFREPKQFPFMQDGGSGLGAARHDLLRPFSSPEDGANITTTRSACNGVPHGLDPECALSLLSSSLHPSPAAGISSATAPPQFAPSSFSRIAASSQAVTTAFASDGGSVAGDHVLVPAVTYEDPSQAMPFSW

QV

>AtSBP3

MSMRRSKAEGKRSLRELSEEEEEEEETEDEDTFEEEEALEKKQKGKATSSSGVCQVESCTADMSKAKQYH

KRHKVCQFHAKAPHVRISGLHQRFCQQCSRFHALSEFDEAKRSCRRRLAGHNERRRKSTTD

>OsSBP8

MMNVPSAAAASSCDDFGYNATPPPPPSLLPIMDQDGGGGSIQRDHHQHHNHQQLGYNLEPSSLALLPPSN

AAAAAAHHATIAHASPHDLLQFYPTSHYLAAAGGAGGGGNPYSHFTAAAAAGSTFQSYYQQPPQDAPEYY

FPTLVSSAEENMASFAATQLGLNLGYRTYFPPRGGYTYGHHPPRCQAEGCKADLSSAKRYHRRHKVCEHH

SKAPVVVTAGGLHQRFCQQCSRFHLLDEFDDAKKSCRKRLADHNRRRRKSKPSDGEHSGEKRRAQANKSA

ATKDKAGSSSKNAGIGDGFETQLLGGAHMSKDQDQAMDLGEVVKEAVDPKGKASMQQQQQQAHHGIHQQSHQQHGFPFPSSSGSCLFPQSQGAVSSTDTSNIAQVQEPSLAFHQQHHQHSNILQLGQAMFDLDFDH

>OsSBP5

MAVPAAAVAADVVDFGYAAPMPPPYVGFDPAGMGGERQLFQHGGACHGLYDGGLDFSAAAAFQEAATLGV

GLPGGNLLQSLAPPAAAAATPSSLQMPMMMSLPGLPATAADVYPFGGGGFVKREDGPVLDVVGGGGGGRI

GLNLGRRTYFSPADVLAVDRLLLRSRLGGMGMEMGMGMGVLGLGLAAAAHHHQPPRCQAEGCKADLSAAK

HYHRRHKVCDFHAKAAAVLAAGKQQRFCQQCSRFHVLAEFDEAKRSCRKRLTEHNRRRRKPTAGGQSSKD

SPPPPPSKKGTDASIASSYTSCDHHKAAASTTTASGVSCLQELADHHDVGGGHQAAMAAAPPPTLSLAAL

PPQEEDDEDEDGGLGNVLMMQQHHQRRRLQHDGDGDDDVAAAAAHHHLMRSLARQQQQHRHSSGCSNNNDGDDDDHNNNNNILSCSSASDQQNSSNNNNMHFFEVDFI

>AtSBP16

MGELPKDDWQMNRWKWDGQRFEAIELQGESLQLSNKKGLDLNLPCGFNDVEGTPVDLTRPSKKVRSGSPG

SGGGGGGNYPKCQVDNCKEDLSIAKDYHRRHKVCEVHSKATKALVGKQMQRFCQQCSRFHLLSEFDEGKR

SCRRRLDGHNRRRRKTQPDAITSQVVALENRDNTSNNTNMDVMALLTALVCAQGRNEATTNGSPGVPQRE

QLLQILNKIKALPLPMNLTSKLNNIGILARKNPEQPSPMNPQNSMNGASSPSTMDLLAALSASLGSSAPE

AIAFLSQGGFGNKESNDRTKLTSSDHSATTSLEKKTLEFPSFGGGERTSSTNHSPSQYSDSRGQDTRSSL

SLQLFTSSPEEESRPKVASSTKYYSSASSNPVEDRSPSSSPVMQELFPLHTSPETRRYNNYKDTSTSPRT

SCLPLELFGASNRGATANPNYNVLRHQSGYASSGSDYSPPSLNSNAQERTGKISFKLFEKDPSQLPNTLR

TEIFRWLSSFPSDMESFIRPGCVILSVYVAMSASAWEQLEENLLQRVRSLVQDSEFWSNSRFLVNAGRQL

ASHKHGRIRLSKSWRTLNLPELITVSPLAVVAGEETALIVRGRNLTNDGMRLRCAHMGNYASMEVTGREH

RLTKVDELNVSSFQVQSASSVSLGRCFIELENGLRGDNFPLIIANATICKELNRLEEEFHPKDVIEEQIQ

NLDRPRSREEVLCFLNELGWLFQRKWTSDIHGEPDFSLPRFKFLLVCSVERDYCSLIRTVLDMMVERNLG

KDGLLNKESLDMLADIQLLNRAIKRRNTKMAETLIHYSVNPSTRNFIFLPSIAGPGDITPLHLAASTSSS

DDMIDALTNDPQEIGLSCWNTLVDATGQTPFSYAAMRDNHSYNTLVARKLADKRNGQISLNIENGIDQIG

LSKRLSSELKRSCNTCASVALKYQRKVSGSRRLFPTPIIHSMLAVATVCVCVCVFMHAFPMVRQGSHFSW

GGLDYGSI

>AtSBP8

MLDYEWDNPSSIVLSGDERNPDSDPTRSSFSFFDPISHYNNDHRHITISPPLLSSFSNQQQQHHLTLYGQ

TNSNNQFLHHHHHHHSLYGSTTTTTPYGASDPIYHPHSSAPPASLFSYDQTGPGSGSGSSYNFLIPKTEV

DFTSNRIGLNLGGRTYFSAADDDFVSRLYRRSRPGESGMANSLSTPRCQAEGCNADLSHAKHYHRRHKVC

EFHSKASTVVAAGLSQRFCQQCSRFHLLSEFDNGKRSCRKRLADHNRRRRKCHQSASATQDTGTGKTTPK

SPNDSGVKASSSPSSNAPPTISLECFRQRQFQTTASSSTSASSSSNSMFFSSG

>OsSBP16

MEWDLKMPPAASWELADELENSGGGGVPAAVSSSSAAVGGGVNAGGGGRQECSVDLKLGGLGEFGGGGAQPRVAVAGEPAKGKGPAAAATGAAAAASSAPAKRPRGAAAAGQQQCPSCAVDGCKEDLSKCRDYHRRHKVC

EAHSKTPLVVVSGREMRFCQQCSRFHLLQEFDEAKRSCRKRLDGHNRRRRKPQPDPMNSASYLASQQGAR

FSPFATPRPEASWTGMIKTEESPYYTHHQIPLGISSRQQHFVGSTSDGGRRFPFLQEGEISFGTGAGAGG

VPMDQAAAAAAASVCQPLLKTVAPPPPPHGGGGSGGGKMFSDGGLTQVLDSDCALSLLSAPANSTAIDVG

GGRVVVQPTEHIPMAQPLISGLQFGGGGGSSAWFAARPHHQAATGAAATAVVVSTAGFSCPVVESEQLNT

VLSSNDNEMNYNGMFHVGGEGSSDGTSSSLPFSWQ

>OsSBP4

MDWMPPPKPTSPRSPPLLWDWADAAVPGSSSGEVSAAAAAAAAHPGRRRKEKRGRAEEGGGGGGEVRCQVEGCGVELVGVKDYHRKHRVCEAHSKFPRVVVAGQERRFCQQCSRFHALSEFDQKKRSCRRRLYDHNARRR

KPQTDVFSYASARPPSSLLFDDNRQISFVWNKAPLSHVRPFAISPWESSSEVGTTDGHIYLDKSHISKSL

PAFNTDIDELLPMKDFSAATIWMFFGVLFIRTAQESCMSFH

>AtSBP9

MEMGSNSGPGHGPGQAESGGSSTESSSFSGGLMFGQKIYFEDGGGGSGSSSSGGRSNRRVRGGGSGQSGQ

IPRCQVEGCGMDLTNAKGYYSRHRVCGVHSKTPKVTVAGIEQRFCQQCSRFHQLPEFDLEKRSCRRRLAG

HNERRRKPQPASLSVLASRYGRIAPSLYENGDAGMNGSFLGNQEIGWPSSRTLDTRVMRRPVSSPSWQIN

PMNVFSQGSVGGGGTSFSSPEIMDTKLESYKGIGDSNCALSLLSNPHQPHDNNNNNNNNNNNNNNTWRAS

SGFGPMTVTMAQPPPAPSQHQYLNPPWVFKDNDNDMSPVLNLGRYTEPDNCQISSGTAMGEFELSDHHHQ

SRRQYMEDENTRAYDSSSHHTNWSL

>OsSBP10

MMSGRMNAAGDESPFPFGAMQAPGPGAYVGFDHGAAAVAAAAAAAQRAGMLQHHHHHMYDGLDFAAAMQFGGGQDAPPHPQLLALPPSMAAPPPPPMPMPLQMPMTMPMPGDVYPALGIVKREGGGGGQDAAAGRIGLNLGRRTYFSPGDMLAVDRLLMRSRLGGVFGLGFGGAHHQPPRCQAEGCKADLSGAKHYHRRHKVCEYHAKASVVAASGKQQRFCQQCSRFHVLTEFDEAKRSCRKRLAEHNRRRRKPAAAATTAVAAAKDAAAAPVAAGKKPSGGAATSYTGDNKNVVSMSAAKSPISSNTSVISCLPEQGKHAAAAARPTALTLGGAPPHESSAPQIGAML

HHHHHHQQDHMQVSSLVHINGGGGGGSNNILSCSSVCSSALPSTATNGEVSDQNNDNSHNNGGNNNNMHLFEVDFM

>OsSBP17

MATGGSGGGGGGGGGGDDVHGLKFGKKIYFEQDAAASASAAAVESSSTSSGGGGKKGKGVAAAAAPPPPL

PPRCQVEGCGVDLSGVKPYYCRHKVCYMHAKEPIVVVAGLEQRFCQQCSRCSVHMVRFHQLPEFDQEKKS

CRRRLAGHNERRRKPTPGPLSSRYGRLAASFHEEPGRSRSFVVDFSYPRVPSSVRDAWPAIQPSDRMSGS

IQWQGGHELHPHRSAVAGYSDHHAFSSHGGSAAGAPMLHHPAFELTSGGCLAGVATDSSCALSLLSTQPW

DTTQSTSSHNRSPPMSSTASAFGGGNNPVSPSVMASNYMAASPGWNSSSRGHDGARNVHLPPPHGVVLNE

VPPGSVHHGHFSGELELALQGGAPSNRPEAEHGSGSGAFSHSTNAMNWSL

>AtSBP6

MDSWSYGRSVFMSNETLLPCDTFAKNRRFEQRLSNNDDVLISDMAGNSNGFSAVSITKVVPEEEDEENIS

SSSKFSSQELNRIDFKLRSFLDLGNDDDDTSSRGFALPSKKSRASNLCSQNPLCQVYGCSKDLSSSKDYH

KRHRVCEAHSKTSVVIVNGLEQRFCQQCSRFHFLSEFDDGKRSCRRRLAGHNERRRKPAFYFLPGKRHKL

LRTSQDVVGNKFLENSSLVLPESFPGSLLYRVIDEDDHRTSRLVSFKDEPTCSMFPTNEQNSSRTYESKP

AIYSTEVSSIWDLHETAASRSTRALSLLSAQSQQHLSKFPNTTFSITQPNQNLNHSSSTDYHQMEQPLWI

DPGKTNSAGSSSCKGKGTSTVDLLQLSSHLQRIEQQRNYTGDVKQEYNELYFPGS

>OsSBP15

MQREVGPQVAPPMFLHQIQPLPPHATAAKKRGNPWPAAAVAAAEAKGGGNWNPRMWDWDSRALTAKPSSDALRVNAGLSHHQQQQQQSPPAAAKAAEALRQGGGGSGGLNLQLGLREDAATPMDVSPAATTVSSSPSPPASSAPAQEPVVRPSKRVRSGSPGSASGGGGGGGGGGNSGGGGGSYPMCQVDDCRADLTNAKDYHRRHKVCEIHGKTTKALVGNQMQRFCQQCSRFHPLSEFDEGKRSCRRRLAGHNRRRRKTQPTDVASQLLLPGNQENAA

NRTQDIVNLITVIARLQGSNVGKLPSIPPIPDKDNLVQIISKINSINNGNSASKSPPSEAVDLNASHSQQ

QDSVQRTTNGFEKQTNGLDKQTNGFDKQADGFDKQAVPSTMDLLAVLSTALATSNPDSNTSQSQGSSDSS

GNNKSKSQSTEPANVVNSHEKSIRVFSATRKNDALERSPEMYKQPDQETPPYLSLRLFGSTEEDVPCKMD

TANKYLSSESSNPLDERSPSSSPPVTHKFFPIRSVDEDARIADYGEDIATVEVSTSRAWRAPPLELFKDS

ERPIENGSPPNPAYQSCYTSTSCSDHSPSTSNSDGQDRTGRIIFKLFGKEPSTIPGNLRGEIVNWLKHSP

NEMEGYIRPGCLVLSMYLSMPAIAWDELEENLLQRVNTLVQGSDLDFWRKGRFLVRTDAQLVSYKDGATR

LSKSWRTWNTPELTFVSPIAVVGGRKTSLILKGRNLTIPGTQIHCTSTGKYISKEVLCSAYPGTIYDDSG

VETFDLPGEPHLILGRYFIEVENRFRGNSFPVIIANSSVCQELRSLEAELEGSQFVDGSSDDQAHDARRL

KPKDEVLHFLNELGWLFQKAAASTSAEKSDSSGLDLMYFSTARFRYLLLFSSERDWCSLTKTLLEILAKR

SLASDELSQETLEMLSEIHLLNRAVKRKSSHMARLLVQFVVVCPDDSKLYPFLPNVAGPGGLTPLHLAAS

IEDAVDIVDALTDDPQQIGLSCWHSALDDDGQSPETYAKLRNNNAYNELVAQKLVDRKNNQVTIMVGKEE

IHMDQSGNVGEKNKSAIQALQIRSCNQCAILDAGLLRRPMHSRGLLARPYIHSMLAIAAVCVCVCVFMRA

LLRFNSGRSFKWERLDFGTI

>OsSBP7

MEGNGCGGSGATPRGVVGMHWAPVVTSPPSPQPPFLPPAPCRPDVQMQQQGGLTCLKLGKRPCFWGGDGAGQVAQGSGGGGGGGGGGSADQGKRKEKAATAVPVVPRCQVEGCDITLQGVKEYHRRHKVCEVHAKAPRVVVHGTEQRFCQQCSRFHVLAEFDDAKKSCRRRLAGHNERRRRSNASEAMARGSAHPHGMPVLGHGFPPYGL

PTSSAGALSLLSSARATGPWLMPTPDISARSSAALDELIAENRAALLSWQFFSDRQPPPAGRPTGRSPGS

ETAGGWHAHLQARPPPPGAGGQHENQSGHVTLDLMQATTAAGGSGAPFRPVPARPPKEGGDAGCTSDAWTPSPMEGARVV

>AtSBP12

MEARIEGEVEGHSLEYGFSGKRSVEWDLNDWKWNGDLFVATQLNHGSSNSSSTCSDEGNVEIMERRRIEM

EKKKKRRAVTVVAMEEDNLKDDDAHRLTLNLGGNNIEGNGVKKTKLGGGIPSRAICCQVDNCGADLSKVK

DYHRRHKVCEIHSKATTALVGGIMQRFCQQCSRFHVLEEFDEGKRSCRRRLAGHNKRRRKANPDTIGNGT

SMSDDQTSNYMLITLLKILSNIHSNQSDQTGDQDLLSHLLKSLVSQAGEHIGRNLVGLLQGGGGLQASQN

IGNLSALLSLEQAPREDIKHHSVSETPWQEVYANSAQERVAPDRSEKQVKVNDFDLNDIYIDSDDTTDIE

RSSPPPTNPATSSLDYHQDSRQSSPPQTSRRNSDSASDQSPSSSSGDAQSRTDRIVFKLFGKEPNDFPVA

LRGQILNWLAHTPTDMESYIRPGCIVLTIYLRQDEASWEELCCDLSFSLRRLLDLSDDPLWTDGWLYLRV

QNQLAFAFNGQVVLDTSLPLRSHDYSQIITVRPLAVTKKAQFTVKGINLRRPGTRLLCTVEGTHLVQEAT

QGGMEERDDLKENNEIDFVNFSCEMPIASGRGFMEIEDQGGLSSSFFPFIVSEDEDICSEIRRLESTLEF

TGTDSAMQAMDFIHEIGWLLHRSELKSRLAASDHNPEDLFSLIRFKFLIEFSMDREWCCVMKKLLNILFE

EGTVDPSPDAALSELCLLHRAVRKNSKPMVEMLLRFSPKKKNQTLAGLFRPDAAGPGGLTPLHIAAGKDG

SEDVLDALTEDPGMTGIQAWKNSRDNTGFTPEDYARLRGHFSYIHLVQRKLSRKPIAKEHVVVNIPESFN

IEHKQEKRSPMDSSSLEITQINQCKLCDHKRVFVTTHHKSVAYRPAMLSMVAIAAVCVCVALLFKSCPEV

LYVFQPFRWELLEYGTS
