# Supplementary material for: The maize gene ZmSBP17 encoding an SBP transcription factor confers osmotic resistance in transgenic Arabidopsis
Source: Front Plant Sci. 2024 Nov 7;15:1483486. doi: 10.3389/fpls.2024.1483486 (PMC11578699; doi:10.3389/fpls.2024.1483486)
Supplement: Supplementary File 3 — Identification of the recombinant plasmid pGBKT7-ZmSBP17. [file Table3.docx]

Supplemental file 4. Cis-elements in the *ZmSBP17* promoter related to abiotic stress

| **Cis-elements** | **Core sequences** | **Function** |
| --- | --- | --- |
| ABRE | ACGTG  GCAACGTGTC  TACGGTC | Cis-acting elements involved in abscisic acid responsiveness |
| ARE | AAACCA | A cis-acting regulatory element essential for anaerobic induction |
| LTR | CCGAAA | A cis-acting element involved in low-temperature responsiveness |
| MBS | CAACTG | MYB-binding site involved in salt and drought stress |
