# Supplementary material for: The maize gene ZmSBP17 encoding an SBP transcription factor confers osmotic resistance in transgenic Arabidopsis
Source: Front Plant Sci. 2024 Nov 7;15:1483486. doi: 10.3389/fpls.2024.1483486 (PMC11578699; doi:10.3389/fpls.2024.1483486)
Supplement: Supplementary File 6 — qRT-PCR analysis of ZmSBP17 in maize plants under salt and PEG stress. [file Table6.docx]

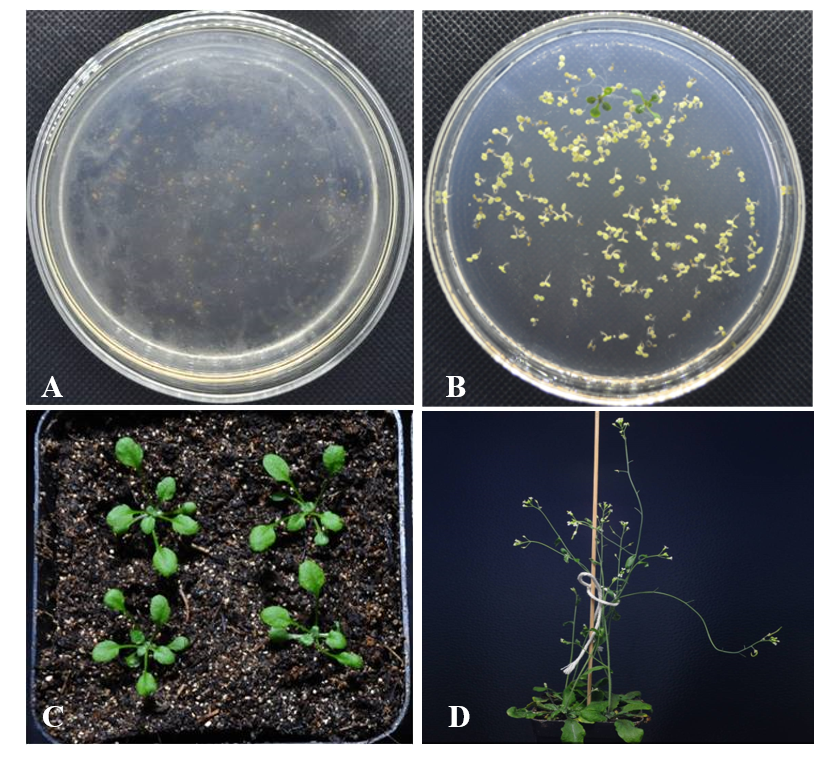


**Transformation process diagram**

**Note:** A, refers to sowing T_1_ generation *Arabidopsis* seeds in 1/2 MS medium, B refers to the screening results of T_1_ generation kana resistance, C refers to transplanting T_1_ generation transgenic *Arabidopsis*, and D refers to harvesting T_1_ generation *Arabidopsis* seeds


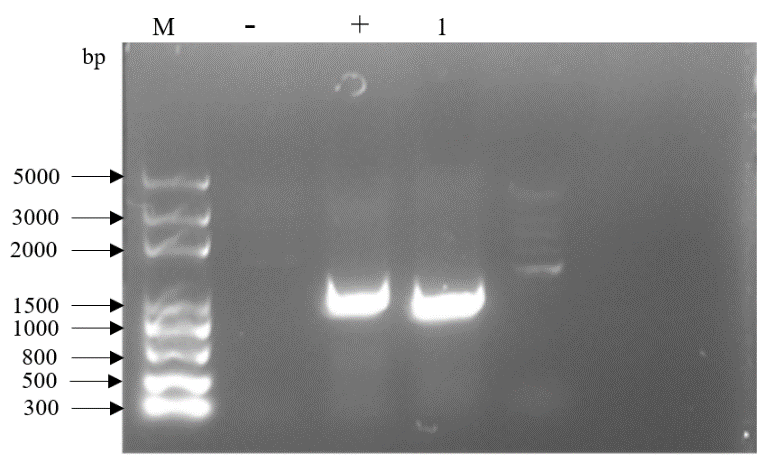


**Verification of T_1_ positive plants by PCR**

**Note: A**, *Arabidopsis* genomic DNA electrophoresis; B, PCR verification electrophoresis of T_1_ generation positive plants; M, DNA marker DL5000; -, Negative control; +, Positive control; 1, It is the PCR band of *Arabidopsis* positive plants infected by *ZmSBP17* gene.
