# Supplementary material for: The maize gene ZmSBP17 encoding an SBP transcription factor confers osmotic resistance in transgenic Arabidopsis
Source: Front Plant Sci. 2024 Nov 7;15:1483486. doi: 10.3389/fpls.2024.1483486 (PMC11578699; doi:10.3389/fpls.2024.1483486)
Supplement: Supplementary File 7 — Overexpression of ZmSBP17 in Arabidopsis thaliana. [file DataSheet1.docx]

**
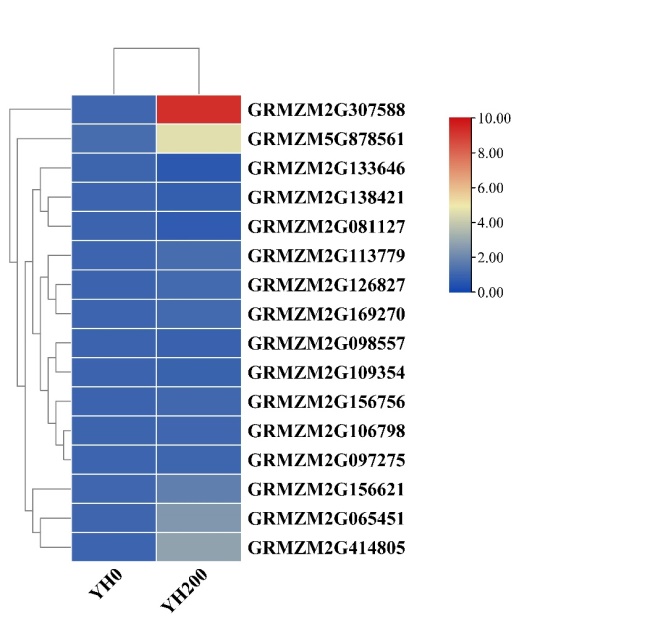
**

qRT-PCR analysis of SBP family genes

note: After 200 mmol/L NaCl stress, only 16 genes in the 31 maize ZmSBPs family genes were detected to have expression levels, and only the *GRMZM2G307588* (*ZmSBP17*) and *GRMZM5G878561* (*ZmSBP22*) genes were significantly upregulated, while the expression levels of other genes were low or not significant.


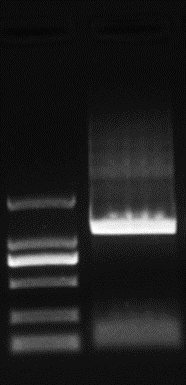


M 1

**The sequence of *ZmSBP17* was isolated by gel electrophoresis**

Marker：2000 bp, 1000 bp, 750 bp, 500 bp, 250 bp, 100 bp

The Sequencing Analysis of *ZmSBP17*

**
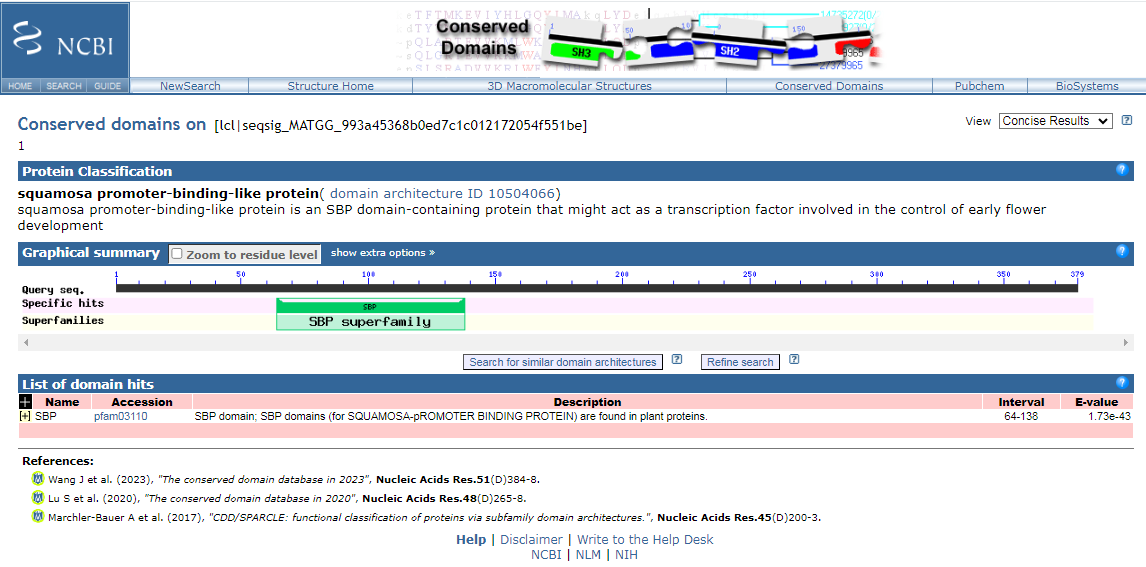
**

**Conserved domain of ZmSBP17**
