# Supplementary material for: The maize gene ZmSBP17 encoding an SBP transcription factor confers osmotic resistance in transgenic Arabidopsis
Source: Front Plant Sci. 2024 Nov 7;15:1483486. doi: 10.3389/fpls.2024.1483486 (PMC11578699; doi:10.3389/fpls.2024.1483486)
Supplement: Supplementary File 8 — Germination rates of OE and WT seeds under salt or PEG stress and activities of ROS-scavenging enzymes. [file DataSheet2.docx]

**
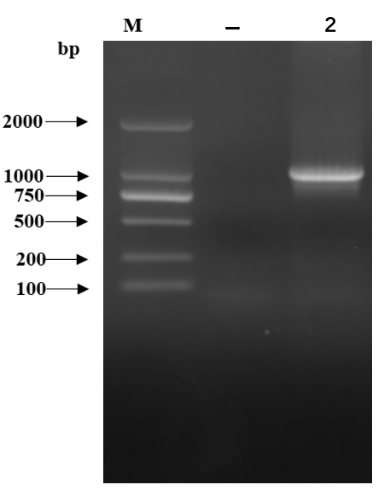

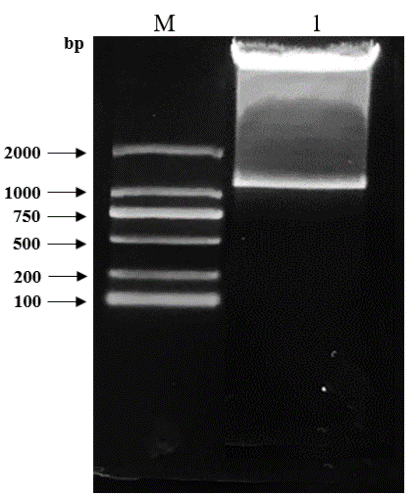
**

**The analysis of PCR and enzyme digestion verification of recombinant plasmid pGBKT7-*ZmSBP17***

Note：M: DNA Marker DL 2000；-：阴性对照； 1：重组质粒双酶切 2：重组质粒菌液PCR

**
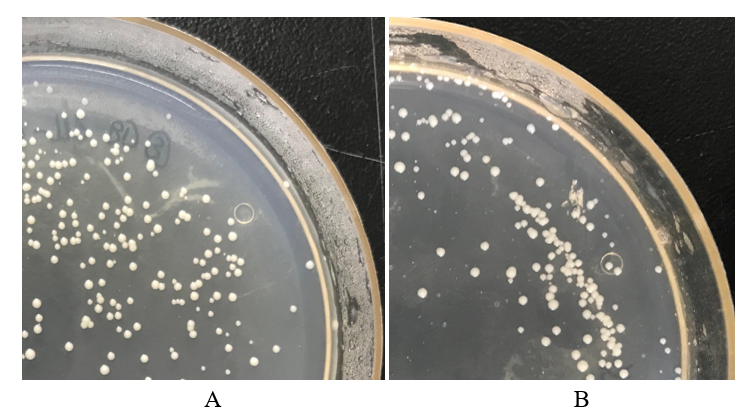
**

**Toxicity identification of bait vector pGBKT7- *ZmSBP17***

Note：A: The growth of bait vector pGBKT7- *ZmSBP17* on SD/-Trp medium; B: The growth of empty vector pGBKT7 on SD/-Trp medium


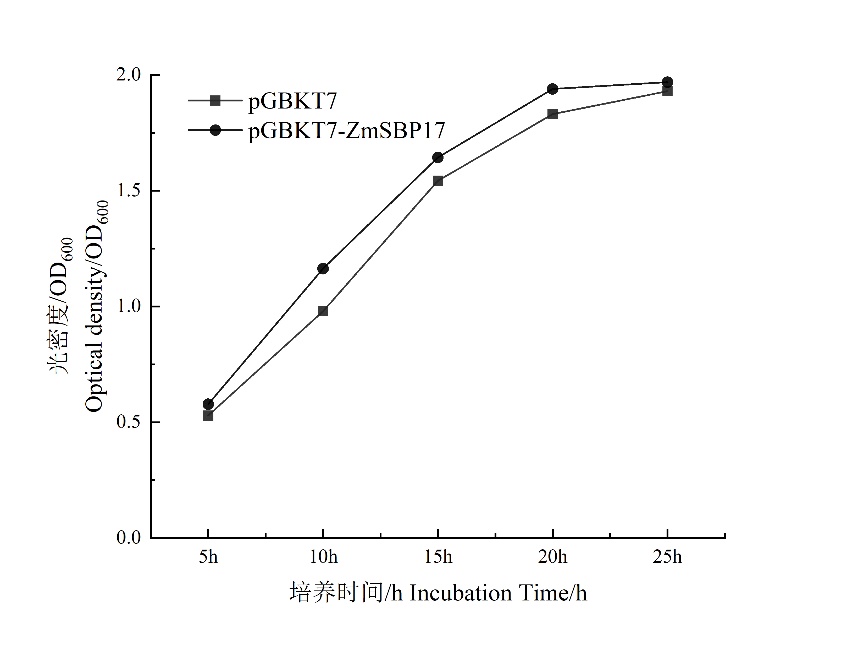


**The growth curve of yeast cells containing plasmids**
